# Progress of autonomic disturbances in narcolepsy type 1

**DOI:** 10.3389/fneur.2023.1107632

**Published:** 2023-03-06

**Authors:** Ying Wang, Qingqing Sun, Qi Tang, Yanan Zhang, Mingyang Tang, Dong Wang, Zan Wang

**Affiliations:** Sleep Center, Department of Neurology, The First Hospital of Jilin University, Changchun, China

**Keywords:** narcolepsy type 1, hypocretin, autonomic nervous system, sympathetic, parasympathetic

## Abstract

Narcolepsy type 1 is a kind of sleep disorder characterized by a specific loss of hypocretin neurons in the lateral hypothalamus and reduced levels of hypocretin-1 in the cerebrospinal fluid. Hypocretin deficiency is associated with autonomic disorders. This article summarizes the autonomic disorders and possible mechanisms associated with narcolepsy type 1. Patients with narcolepsy type 1 often have various systemic autonomic symptoms, including non-dipping blood pressure, reduced heart rate variability, dynamic cerebral autoregulation impairment, reduced gastric motility and emptying, sleep-related erectile dysfunction, skin temperature abnormalities, and blunted pupillary light reflex. Similar findings should strengthen the recognition and intervention of these disturbances in clinical practice. In addition to hypocretin deficiency, current evidence also indicates that pharmacological therapy (including psychostimulants and anti-cataplectic drugs) and comorbidities may contribute to the alterations of autonomic system observed in narcolepsy type 1.

## 1. Introduction

Narcolepsy type 1 (NT1) is a chronic sleep disorder with major clinical manifestations including excessive daytime sleepiness, cataplexy (a sudden loss of muscle tone triggered by strong, mainly positive, emotions), sleep paralysis, hypnagogic hallucinations, and nocturnal sleep disorder (1). Patients can also present with multiple chronic comorbidities including obesity, depressive disorder, migraine, precocious puberty and other sleep disorders (such as rapid eye movement sleep behavior disorder and obstructive sleep apnea) (2). The most significant neuropathological change is the selective and irreversible loss of hypocretin-producing neurons in the lateral hypothalamus (3). Hypocretin neuropeptides consisted of hypocretin-1 (hcrt-1) and hypocretin-2 (hcrt-2), and they modulate their actions *via* hcrt-1 and hcrt-2 receptors. Patients with NT1 often have low levels of hcrt-1 in the cerebrospinal fluid.

**Figure 1:**
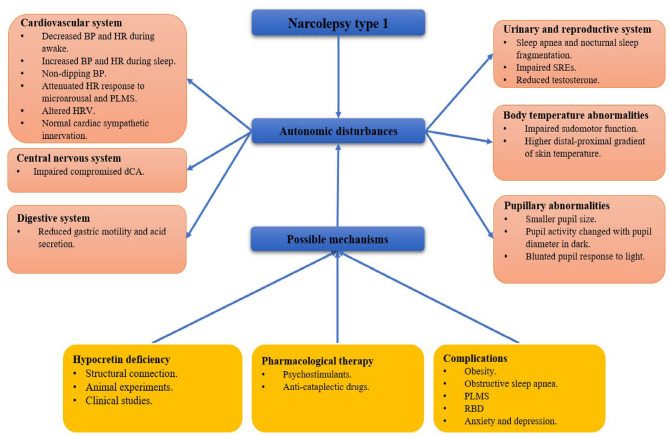
A framework of autonomic disturbances and possible mechanisms in narcolepsy type 1. BP, blood pressure; HR, heart rate; PLMS, period limb movements during sleep; HRV, heart rate variability; dCA, dynamic cerebral autoregulation; SRE, sleep-related erection; RBD: rapid eye movement sleep behavior disorder.

Hypocretin neurons have widespread projections to different areas involved in regulating the sleep-wake cycle, energy metabolism, neuroendocrine, body temperature, and cardiovascular functions, which are associated with changes in the autonomic nervous system (4). Clinically, autonomic dysfunction often affects visceral organs, vascular smooth muscle, myocardium, and glands activities (5). However, autonomic symptoms are easily ignored compared with the typical symptoms in NT1. Here, we review the autonomic disorders and their possible mechanisms in patients with NT1 (Figure 1).

### 1.1. Autonomic disorders

#### 1.1.1. Cardiovascular system

##### 1.1.1.1. Changes in blood pressure and heart rate

Previous studies suggested that 52% of adults with NT1 and 37% of children with NT1 and other hypersomnia disorders had the symptoms of orthostatic intolerance, indicating impairment of cardiovascular autonomic regulation (5, 6). Autonomic disorders in the cardiovascular system may increase the risk of cardiovascular events and reduce the quality of life of patients with NT1.

Several studies on cardiovascular changes have focused on daytime wakefulness, different sleep stages, and wake-sleep transitions. Donadio et al. (7) demonstrated lower heart rate (HR), blood pressure (BP), and resting muscle sympathetic nerve activity using direct microneurographic recordings in patients with NT1 during wakefulness. In contrast, other studies found a significant increase in HR in patients with NT1 vs. controls during wakefulness, and the non-rapid eye movement (NREM) and rapid eye movement (REM) stages (8–10). In addition, it is generally believed that patients with NT1 are prone to have non-dipping blood pressure, defined as a nocturnal BP decrease < 10% of the daytime BP (8, 11, 12). Dauvilliers et al. (13) reported a higher percentage of non-dipping BP in NT1 compared to healthy controls (31 vs. 3%). Grimaldi et al. (8) observed a significantly increased systolic BP in 10 untreated patients with NT1 vs. controls during nighttime REM sleep.

In summary, BP and HR were lower during resting wakefulness but higher during nighttime sleep in patients with NT1. Impaired cardiovascular regulation ability is not beneficial for the maintenance of normal physiological functions. Furthermore, microarousal and periodic leg movement events during sleep (PLMS) may affect BP and HR. Two previous studies found that the amplitude of microarousal and PLMS-related HR responses was significantly reduced in patients with NT1 compared to controls, suggesting poor cardiac autonomic nervous regulation (14, 15).

##### 1.1.1.2. Measurement of cardiovascular autonomic disorders

Heart rate variability is widely used to evaluate autonomic changes, including frequency-domain, time-domain, and non-linear correction analysis. In the frequency domain, low frequency (LF) is modulated by both the sympathetic (SNS) and parasympathetic (PNS) nervous systems, while high frequency (HF) is only affected by PNS activity.

The LF/HF ratio provides a measure of sympathovagal balance, which generally increases with high SNS activity and decreases with high PNS activity (16). Grimaldi et al. (17) studied heart rate variability in NT1 in the resting supine position and found an increased LF/HF ratio favoring enhanced SNS activity. However, after removing the effect of respiratory frequency on the HF component, there were no significant differences in the LF/HF ratio between NT1 and controls during any sleep stage or wakefulness (9). Silvani et al. (10) observed a significant reduction of cardiac baroreflex sensitivity and time-frequency index [—square root of the mean of the sum of the squares of differences between adjacent normal-to-normal interval (RMSSD)] in NT1 vs. controls during wakefulness before sleep, which reflect the function of cardiac PNS modulation.

^123^I-metaiodobenzylguanidine cardiac scintigraphy is a reliable method for the objective evaluation of cardiac adrenergic nerve activity. Barateau et al. found normal cardiac sympathetic innervation in NT1 by comparing the delayed heart/mediastinum ratio of patients with NT1 to that in control subjects. However, the study did not calculate the early heart/mediastinum ratio and washout rate, with the latter being the most reliable biomarker to reflect cardiac sympathetic nerve activity (18).

Generally, non-dipping BP and decreased heart rate variability indicate that the ability of cardiovascular autonomic regulation is decreased in patients with NT1, making it impossible to better adapt to the changing environment. As for the increase or decrease in SNS and PNS during the wake and sleep stages, there are contradictions between studies, for which possible reasons are the small sample size, insufficient adjustment of confounding factors, and lack of standard measurement methods.

#### 1.1.2. Possible mechanisms of cardiovascular autonomic disorders

##### 1.1.2.1. Hypocretin deficiency

Hypocretin neurons have widespread connectivity with neurons involved in autonomic control, including the paraventricular nucleus of the hypothalamus, homonymous noradrenergic cell groups of the pons, medullary raphe nuclei, rostral ventrolateral medulla, rostral ventromedial medulla, nucleus ambiguus, nucleus of the tractus solitarius, and dorsal motor nucleus of the vagus nerve, which set the foundation for the involvement of hypocretin in autonomic regulation and autonomic disturbances in NT1 (4).

Several animal studies have confirmed the involvement of hypocretin in the regulation of cardiovascular autonomic nervous activity. Machado et al. (19) observed an increase in BP and HR by injecting hcrt-1 into the rostral ventromedial medulla of conscious rats, but no cardiovascular changes were observed following the injection of saline. Shirasaka et al. (20) also found an increase in BP, HR, renal sympathetic nerve activity, and plasma catecholamine levels following intracerebroventricular injection of hcrt-1 in conscious rats. Two animal studies showed that hcrt-1 has an activating effect on the cardiovascular sympathetic nerve, but it is worth noting that hcrt-1 concentration was much higher under experimental conditions than under physiological conditions. Iigaya et al. (21) observed a decrease in BP, HR, and renal sympathetic nerve activity by blocking the hcrt receptor. Three previous studies also showed decreased BP and HR in hypocretin gene-deficient or gene-silent animals by gene knockout, small interfering RNA, and transgenic techniques (22–24).

A clinical study showed that all patients with narcolepsy had a significantly attenuated HR response to arousals and PLMS, particularly patients with NT1, and hcrt-1 deficiency could be an independent predictor of reduced HR response in multivariate linear regression analysis (15). Donadio et al. (7) demonstrated a correlation between cerebrospinal fluid hcrt-1 concentration and HR or muscle sympathetic nerve activity. There is a negative correlation between the pulse transit time and arterial BP, with pulse transit time lengthensing if vessels become less stiff due to a decrease in arterial BP. Vandi studied 27 pediatric patients with NT1 and found a reduced lengthening of pulse transit time during total sleep and REM sleep compared with nocturnal wakefulness, which was more severe in subjects with lower cerebrospinal fluid levels of hcrt-1 (12).

These results support the direct effect of hcrt-1 on autonomic regulation. However, other studies have not supported this conclusion. Barateau et al. (5) reported that a higher “scales for outcomes in Parkinson's disease-autonomic” (SCOPA-AUT) score was not associated with cerebrospinal fluid hcrt-1 levels. In the same year, another study found that a delayed heart/mediastinum value was independent of hcrt-1 (18).

##### 1.1.2.2. Pharmacological therapy

Life-long treatment with psychostimulants and anti-cataplectic drugs can affect the autonomic nervous system of patients with NT1. Bosco et al. found that patients with NT1 treated with psychostimulants had higher 24-h diastolic BP and HR than untreated patients. The prevalence of hypertension was also significantly higher than that in untreated patients. They also found that the combination of anti-cataplectic drugs and psychostimulants showed a synergistic effect on BP (25). The effects of psychostimulants (such as methylphenidate) and anti-cataplectic drugs (such as venlafaxine and fluoxetine) on the autonomic nervous system are related to their sympathomimetic mechanisms of action, including the promotion of presynaptic membrane release of monoaminergic transmitters and inhibition of monoaminergic transmitters reuptake.

##### 1.1.2.3. Comorbidities

Patients with NT1 have a variety of comorbidities such as obesity, obstructive sleep apnea, PLMS, sleep behavior disorder during REM, anxiety, and depression, which are all closely associated with autonomic dysfunction (26–29). Rocchi et al. (30) demonstrated a significant and positive correlation between body mass index and systolic BP in the supine resting position at 3 and 10 min head-up tilt test; therefore, it is speculated that body weight plays an important role in cardiovascular sympathetic tone. Nocturnal sleep fragmentation has been frequently reported in patients with obstructive sleep apnea, PLMS and REM sleep behavior disorder, which affects the autonomic nervous system in NT1 (14, 15, 31, 32). Symptoms of anxiety and depression have been demonstrated to be common among patients with NT1 (33). Barateau et al. (34) suggested that the severity of depressive symptoms was associated with autonomic impairment. Research showed that the component formula of Suanzaoren Tang had anti-anxiety function by reducing hippocamps 5-hydroxytryptamine level in rats (35, 36). These results indicate the link between psychiatric symptoms and autonomic disorders in NT1.

#### 1.1.3. Central nervous system

Cerebral autoregulation is the ability of the brain to maintain adequate cerebral blood flow in the presence of changes to blood or cerebral perfusion pressure. Dynamic cerebral autoregulation (dCA) is used to study transient changes in cerebral blood flow (37). dCA is regulated by the autonomic nervous system; therefore, sympathovagal balance is important to maintain relative stabilization of cerebral blood flow (38–40). Our previous study found that dCA was impaired in patients with NT1, possibly indicating dysfunction of autonomic nerves innervating cerebral vessels. The hypocretin neurons send projections to monoaminergic neurons, including dopamine, norepinephrine, and 5-hydroxytryptamine. A previous study found that hcrt neurons play critical roles in the sleep/wakefulness pathway by regulating monoaminergic transmitter levels (41). Norepinephrine is a sleep autonomic neuromodulating transmitter and 5-hydroxytryptamine is a vasoactive substance that may have potential effects on dCA (42–44). Hypocretin and monoaminergic transmitter reduction or deficiency can lead to impairment of dCA and autonomic dysfunction in patients with NT1 (45).

#### 1.1.4. Digestive system

It has been reported that 88% of untreated adult patients with NT1 have gastrointestinal disturbances, including drooling, early abdominal fullness, constipation, and straining for defecation. These symptoms may be related to vagal nerve dysfunction regulated by hcrt-1 (5).

Previous studies mainly focused on animal models. Jin et al. found that gastric motility and emptying were enhanced by injecting hcrt-1 into the central nucleus of the amygdala of rats, which expresses the hcrt-1 receptor. This effect was abolished by subdiaphragmatic vagotomy. These results show that the amygdala-vagus-stomach pathway may be involved in regulating gastric motility through hcrt-1 (46). In addition, many other nuclei are directly or indirectly involved in the regulation of gastric acid secretion and gastric motility mediated by hypocretin neurons, including the paraventricular nucleus of the hypothalamus, raphe nucleus of the medulla oblongata, ventral tegmental area and nucleus accumbens (46–48).

#### 1.1.5. Urinary and reproductive system

Barateau et al. found that most patients with NT1 (92%) had urinary symptoms, especially nocturia and incomplete bladder emptying (5). Nocturia symptoms may be related to sleep apnea and nocturnal sleep fragmentation, which improve after continuous positive pressure ventilation (49).

Sexual dysfunction has been reported in 48% of men (erection problems) and 81% of women (vaginal lubrication problems) (5). Sleep-related erection (SRE) often occurs during REM sleep. The results from a study in rats suggested that SRE is regulated by the hypothalamus (49). Karacan et al. conducted SRE tests on 28 patients with NT1 and found that 23 of them who were receiving methylphenidate and imipramine therapy had 20% shorter SREs and incomplete erection, and only two of the other five untreated patients had impaired SRE. These results suggest that sexual dysfunction may be related to the use of stimulants and antidepressants (50). In addition, insufficient testosterone and abnormal hypothalamic-pituitary-gonadal axis activity may be related to male sexual dysfunction. Joshi et al. (51) reduced the level of testosterone in the serum by injecting an hcrt-1 receptor antagonist into adult mice to prove its involvement in sex hormone synthesis. In a study comparing serum gonadotropin levels in males with NT1, pulsatile luteinizing hormone release was diminished compared to controls, indicating that hcrt-1 is involved in the regulation of hypothalamic-pituitary-gonadal axis activity (52).

### 1.2. Other autonomic disorders

#### 1.2.1. Body temperature abnormalities

Up to 87% of patients with NT1 have symptoms related to abnormal thermoregulation, including daytime hyperhidrosis during the day and heat intolerance (5). Abnormal sweat gland function can be assessed using the sudomotor function test. Rocchi et al. (30) found lower hand sudomotor activity significantly in patients with NT1, suggesting an impairment in cholinergic sympathetic activity. However, the results conflict with the clinical symptoms of hyperhidrosis. Other techniques, such as sympathetic skin response and quantitative sudomotor axon reflex tests, are needed to verify this discrepancy.

Previous studies have focused on altered distal and proximal skin temperatures and their relationship with clinical symptoms and sleep architecture. Fronczek et al. measured the skin temperature in 15 untreated patients with NT1 throughout the day and found an increased distal skin temperature and a decreased proximal skin temperature, resulting in a higher gradient. This change is indicative of decreased distal sympathetic vasoconstrictor tone and increased distal skin blood flow in NT1, which may ultimately be attributed to hypocretin deficiency (53).

Skin temperature dysfunction in patients with NT1 is associated with their two core symptoms: excessive daytime sleepiness and nocturnal sleep disorder. Influencing distal skin temperature increased daytime alertness and time of wakefulness (53, 54). An elevated distal-proximal gradient of skin temperature to some extent lead to an increase in slow wave and REM sleep and a decrease in wakefulness, which is helpful in improving the quality of nighttime sleep (55). Vander Heide and colleagues reported that the greater the distal and distal-proximal gradient of skin temperature before daytime sleep episodes, the more likely patients with NT1 were to fall asleep, indicating a strong predictive value of increased distal and distal-proximal gradients of skin temperature for daytime sleep episodes in patients with NT1 (56).

#### 1.2.2. Pupillary abnormalities

Pupillomotor symptoms with increased sensitivity to bright light were observed in 64.2% of patients with NT1 (5). The pupil size is influenced by the degree of arousal, and hcrt-1 is an important neurotransmitter that maintains alertness. Pressman et al. observed that the mean pupillary diameter was significantly smaller in patients with NT1 compared to controls. Pupil activity was correlated with pupil diameter in dark conditions, with maximum pupil size at the highest ratings of alertness and minimum at the lowest alert level (57). Zhou et al. (58) blunted the pupillary response to light by intravitreal injection of an hcrt-1 receptor antagonist in mice, while enhancing the pupil response to light by injection of hcrt-1. It is evident that hcrt-1 plays a role in regulating pupil size and changes, and the clinical symptoms of pupillary abnormalities may be related to reduced pupil diameter and a blunted light response.

## 2. Conclusion

Patients with NT1 often have various clinical autonomic symptoms, but relevant epidemiological studies are still lacking. The SCOPA-AUT questionnaire has been validated for Parkinson's disease, and the feasibility of the subjective tool to assess the severity of autonomic symptoms in NT1 needs further investigation. With more clinical attention being paid to autonomic symptoms, standard objective measurement methods need to be developed. In addition, hypocretin reduction or deficiency alone cannot explain the extent of autonomic disorders in patients with NT1. Finally, it is generally accepted that prolonged autonomic disorders could increase the risk of cardiovascular disease in patients with NT1, and long-term follow-up is necessary in the future.

## Author contributions

QT and YZ were involved in the retrieval of literature and collected the data. YW wrote the initial manuscript. QS, MT, and DW drafted the Figure 1. QS redesigned and revised the manuscript. ZW designed and approved the final version of the manuscript. All authors have read the final manuscript and approved it for submission.
